# Increased attachment security is related to early therapy drop-out in substance use disorders

**DOI:** 10.1186/s13104-018-3251-7

**Published:** 2018-02-20

**Authors:** J. Fuchshuber, M. Hiebler-Ragger, K. Ragger, A. Rinner, H. P. Kapfhammer, H. F. Unterrainer

**Affiliations:** 1 Department of Psychiatry and Psychotherapeutic Medicine, Medical University Graz, Graz, Austria; 2 Center for Integrative Addiction Research, Grüner Kreis Society, Vienna, Austria; 3 Department of Religious Studies, University of Vienna, Schenkenstraße 8-10/5th Floor, 1010 Vienna, Austria

**Keywords:** Attachment styles, Cluster analysis, Substance use disorder, Therapeutic community, Treatment adherence

## Abstract

**Objectives:**

Previous research work suggests a positive association between secure attachment and increased therapy adherence (TA) in different patient groups. However, there is still a strong need for research focusing on the influence of attachment on TA in substance use disorder (SUD) treatment. Hence, this study attempts to investigate the predictive value of different attachment patterns concerning TA in SUD inpatients.

**Results:**

122 (34 female) SUD inpatients completed the Attachment Style Questionnaire (ASQ) during the entry phase of therapeutic community treatment. After 6 weeks, subjects who remained in therapy (*n* = 47) completed the ASQ for a second time. In line with the literature, agglomerative Cluster Analysis suggested a two-cluster solution (Cluster I: increased secure attachment pattern; Cluster II: increased insecure attachment pattern). Notably, inpatients in Cluster I were more likely to drop out of treatment within the first 6 weeks (*p* < .001). Furthermore, subjects showed less “Confidence in Self and Others” (*p* < .05) after 6 weeks of treatment. Our findings indicate a negative predictive value of increased attachment security for TA in SUD inpatients. This finding probably mirrors a more realistic kind of self-assessment. More generally, the importance of considering attachment styles in SUD treatment is underlined.

## Introduction

Substance use disorders (SUD) have been linked with insecure attachment in several studies [1]. This well-established connection is explained by several characteristics observed in individuals displaying an insecure attachment pattern: dysfunctional attributes such as the increased susceptibility and physiological response to stress, as well as the underuse of social support (in conjunction with inadequate help seeking strategies), lead to a predisposition for the frequent use of psychoactive substances as a futile attempt to regulate affects [2–4].

Research focusing on the relationship between attachment styles and treatment adherence (TA) is relatively sparse, these studies have been conducted mostly on somatic patients treated for diabetes and lupus [5, 6]: the results indicate that insecure attachment, especially dismissing and avoidant styles, correspond to worse treatment outcome and compliance. Insecure attachment might lead to increased difficulties in the relationship between patients and caregivers, due to patients’ inability to accept help from others. A study on the role of attachment in TA in SUD inpatients has found anxious-preoccupied attachment to be a predictor for treatment retention [7]. Moreover, several studies also observed a negative association between TA and parameters such as comorbid personality disorder, cognitive deficits and age [8, 9].

In previous research, we observed a higher amount of insecure attachment in different SUD patient groups in comparison to healthy controls [10]. As an extension to this study [10], we intended to examine the role of attachment styles for TA by investigating this group of SUD inpatients for a second time after 6 weeks. Based on former results we hypothesized increased TA in more securely attached SUD patients.

## Main text

### Methods

#### Participants and procedure

The initial sample consisted of 122 inpatients diagnosed with AUD (F10.2x) (*n* = 66) or poly drug use disorder (PUD) (F19.2x) (*n* = 57) by a licensed psychiatrist according to ICD 10 [11]. As our previous research indicated that patients diagnosed with AUD or PUD do not differ in their attachment style and their personality organization [10], we decided not to further differentiate between AUD and PUD inpatients in the present study. After 6 weeks, the 47 participants remaining in treatment were investigated for a second time. All subjects were abstinent while in treatment. Further inclusion criteria were: no current psychotic symptoms and full command of the German language. Assessment took place at therapeutic facilities of the “Grüner Kreis” society, an Austrian association dedicated to the treatment and rehabilitation of people with SUD.

#### Assessment of clinical characteristics and attachment dimensions

Participants where asked about age, gender and level of education. Current medication and comorbid psychiatric diagnoses were assessed through the facilities’ database.

The Attachment Style Questionnaire (ASQ) [12] is a 40-item questionnaire which measures five dimensions of adult attachment: “Confidence in Self and Others” (15 items), “Discomfort with Closeness” (8 items), “Relationships as Secondary” (7 items), “Need for Approval” (4 items) and “Preoccupation with Relationships” (6 items). Items are rated on a 6-point Likert scale. While higher scores in “Confidence in Self and Others” indicate a more secure attachment, higher scores in the other four scales indicate more insecure attachment facets.

#### Statistical analysis

We conducted agglomerative hierarchical cluster analysis including a squared Euclidian distance interval and Ward’s method to establish a parameter for the different attachment attitudes within the inpatient sample. Furthermore, we performed general linear model multivariate and step-wise hierarchical logistic regression analysis for group comparison and the prediction of treatment drop-out. Additional group comparisons were conducted by means of *χ*^2^ tests. The alpha-level was set to .05.

### Results

#### Cluster analysis of attachment dimensions

All ASQ sub-scales showed satisfying internal consistencies (see Table 1), with the exception of “Discomfort with Closeness”, which was therefore not taken into account for further interpretation.

**Table 1:** Differences between Cluster I (n = 81) and Cluster II (n = 41) in attachment dimensions

Measure	α	Cluster I		Cluster II		*F*_(1,120)_	*p*	*η* ^2^
		*M*	*SD*	*M*	*SD*			
ASQ								
Confidence in Self and Others	0.75	37.69	6.77	20.80	4.95	220.44	.000	.63
Discomfort with Closeness^a^	0.51	17.53	4.57	21.20	5.88	14.34	.000	.11
Need for Approval	0.67	10.45	3.81	13.20	3.85	14.01	.000	.11
Preoccupation with Relationships	0.78	16.10	5.96	18.07	6.19	2.91	.091	.02
Relationships as Secondary	0.83	25.27	7.45	29.88	4.01	13.62	.000	.10

*ASQ* Attachment Style Questionnaire

*α* = Cronbach alpha, ^a^ parameter should not be further interpreted as *α* < .60

In line with de Rick and Vanheule [13], a two-cluster solution could be accepted after performing agglomerative cluster-analysis using all ASQ subscales. As shown in Table 1 Cluster I, “increased secure attachment pattern”, was comprised of inpatients with increased scores in “Confidence in Self and Others” (which equals more secure attachment) and decreased scores in almost all other dimensions representing facets of insecure attachment [11]. Conversely, Cluster II, “increased insecure attachment pattern” consisted of inpatients with lower scores in “Confidence in Self and Others”, in combination with increased scores in most insecure attachment dimensions, with the exception of “Preoccupation with Relationships”.

Furthermore, Cluster I inpatients exhibited significantly more comorbid disorders than Cluster II inpatients ($\chi^{2}{_{(1)}}$ = 13.49, *p* < .001). In comparison to healthy controls [9], Bonferroni corrected post hoc analysis revealed that Cluster I inpatients showed lower scores in “Confidence in Self and Others” (*p* < .001) and higher scores in “Relationships as Secondary” (*p* < .001), but lower scores in “Need for Approval” (*p* < .001) and “Preoccupation with Relationships” (*p* < .001). Cluster II inpatients exhibited lower scores in “Confidence in Self and Others” (*p* < .001) and higher scores in “Relationships as Secondary” (*p* < .01), but did not show any differences (*p* > .05) in “Need for Approval” or “Preoccupation with Relationships” in comparison to control subjects.

#### Demographics and clinical characteristics

Characteristics for both groups, inpatients who remained in treatment for at least 6 weeks (TR; *n* = 47) as well as inpatients who dropped out of treatment during the first 6 weeks (TD; *n* = 75), are displayed in Table 2. Both groups consisted of more male (TR: 83%; TD: 65%) than female participants ($\chi^{2}{_{(1)}}$ = 4.48, *p* < .05), while comorbid psychiatric disorders were less common in TR inpatients (Affective Disorder: 15%, Anxiety Disorder: 2%, Schizophrenia: 4%, Personality Disorder 4%) than in TD (Affective Disorder: 43%, Anxiety Disorder: 7%, Schizophrenia: 3%, Personality Disorder: 1%), ($\chi^{2}{_{(5)}}$ = 13.44, *p* < .05).

**Table 2:** Group differences in demographic and clinical characteristics

Measure	TR (*n* = 47)		TD (*n* = 75)		*F*_(1,120)_	*η* ^2^
	*M*	*SD*	*M*	*SD*		
Age (years)	33.02	10.64	36.83	11.94	3.19*	.03
	*n*	*%*	*n*	*%*	$\chi^{2}{_{(1)}}$	*p*
Sex (female)	8	17	26	35	4.47	.034
Comorbid diagnosis	12	26	40	53	9.13	.003
Psychotropic medication	17	36	39	52	2.91	.088
Education > 12 years	6	13	15	20	1.06	.303
Attachment security	23	49	58	77	10.44	.001

TR = remained in therapy; TD = therapy drop-outs, sex: female = 0, male = 1; comorbidity: 0 = no; 1 = yes; psychotropic medication: 0 = no, 1 = yes; education: 0 = less than 12 years of education, 1 = more than 12 years of education; attachment security: Cluster I (more secure attachment pattern) = 0; Cluster II (insecure attachment pattern) = 1

* p > .05

Finally, we found that TR (77%) were more likely than TD (49%) to belong to Cluster II (increased insecure attachment pattern) ($\chi^{2}{_{(1)}}$ = 10.44, *p* < .001).

#### Attachment predictors of therapy adherence

Within TR inpatients, “Confidence in Self and Others” decreased during the first 6 weeks of treatment (*F*_(1,46)_ = 300.89, *p* < .05, *η*^2^ = .13). No relevant differences were found for any of the other dimensions (*p* > .05).

In hierarchical regression analyses (see Table 3), the final regression model accounts for 18% of the estimated variance in TA: sex was entered as a control variable at Step 1 (B = 0.95, Nagelkerke *R*^*2*^ = .05, *p* < .05), comorbidity at Step 2 (B = − 1.05, Nagelkerke *R*^*2*^ = .12, *p* < .01) and attachment security at Step 3 (B = − 1.10, Nagelkerke *R*^*2*^ = .18, *p* < .01). Therefore, attachment security added approximately 6% of the variance to the predictive validity of the regression model.

**Table 3:** Predictors of treatment adherence after 6 weeks (hierarchical logistic regression modelling; n = 122)

	Variable	B	Model *χ*^*2*^	p value	Nagelkerke *R*^*2*^
Step 1			4.69	.03	0.05
	Sex	0.95		.04	
Step 2			11.16	.01	0.12
	Sex	0.63		.19	
	Comorbidity	− 1.05		.01	
Step 3			17.65	.01	0.18
	Sex	0.79		.12	
	Comorbidity	− 0.70		.12	
	Attachment security	− 1.10		.01	

Sex: female = 0; male = 1; comorbidity: no comorbidity = 0; comorbid disorder = 1; attachment security: Cluster II (insecure attachment pattern) = 0; Cluster I (more secure attachment pattern) = 1

### Discussion

The primary goal of this study was to explore the impact of attachment on TA, for the entry phase of treatment (6 weeks), in a group of SUD inpatients. An Agglomerative Cluster analysis was conducted whereby a two-cluster solution, more secure (Cluster I) versus more insecure (Cluster II) attachment patterns, could be acknowledged. In contrast to our assumptions, Cluster I subjects were more likely to drop out during the entry phase of treatment and also more likely to be diagnosed with a comorbid disorder than participants with a more insecure attachment pattern (Cluster II). Hierarchical regression analysis revealed that the influence of the attachment security concealed the effects of the control variables sex and comorbidity on TA. Moreover, the remaining patients showed a significant decrease in the ASQ subscale “Confidence in Self and Others” at the second point of measurement. No other significant changes in attachment dimensions, assessed at the beginning of treatment, were found after 6 weeks.

The surprising results regarding the influence of attachment on TA, which contradicts previous findings, might be partially explained by a lack of self-reflection within TD inpatients. In this study, a self-report measure of adult attachment was applied, which might be susceptible to distorted self-images and lacks the capacity to detect repressed information [14]. This problem might be more prominent in SUD patients than with patients with solely somatic condition [15]. By further considering the increased rate of comorbidity within the TD and the more secure attached group, it seems possible that the self-reported high attachment security more likely reflects an unrealistic (or idealized) image of the self in relationships with others, characterized by primitive defense mechanism like splitting and denial [16]. Furthermore, our findings could mirror a specific mechanism taking place specifically within therapeutic communities, which represents a threat to narcissistically biased self-images: The high amount of group cohesion within the community might lead to strong cognitive dissonances in vulnerable individuals, which in turn leads to early treatment drop-out [17]. A reduction in narcissism might also provide an explanation for a decreased “Confidence in Self and Others” score after 6 weeks of treatment. This result fits with the general approach of therapeutic community treatment to support patients in developing more conscious attitudes towards interpersonal deficits during their stay within the community [18]. Either way, the outcome of a decreasing “Confidence in Self and Others” might mirror an initial euphoria combined with the experience of being sober after such a long time of severe substance misuse. This exhilaration possibly vanishes after some time spent in the therapeutic community.

### Conclusions

In conclusion, our highly preliminary results indicate that caregivers should be especially attentive with patients exhibiting a high amount of comorbidity in combination with a seemingly secure attachment attitude, especially if attachment was captured via standardized self-report measures. However, in line with the literature [5–7], this study confirms the importance of considering the attachment dimension for the treatment of SUD.

### Limitations

- A rather small sample size and the use of a self-report measure for adult attachment styles.
- The focus was only on the first short treatment period of 6 weeks. For the description of long-term treatment effects within a therapeutic community setting further research might focus on an enhanced time-span.
- The study examined a convenience sample. It is necessary to replicate these highly preliminary findings in future studies using randomized controlled trials.
- For a more in depth understanding of our results, further research might especially focus on attachment based therapeutic interventions on TA.

## Abbreviations

ASQ: Attachment Styles Questionnaire
AUD: alcohol use disorder
PUB: poly drug use disorder
SUD: substance use disorders
TD: patients, who dropped out of treatment
TR: patients, who remained in treatment
TA: therapy adherence
